# 20 (S)-Protopanaxadiol Alleviates DRP1-Mediated Mitochondrial Dysfunction in a Depressive Model In Vitro and In Vivo via the SIRT1/PGC-1α Signaling Pathway

**DOI:** 10.3390/molecules29215085

**Published:** 2024-10-28

**Authors:** Pengli Guo, Zixian Wang, Li Sun, Zhongmei He, Jianming Li, Jianan Geng, Ying Zong, Weijia Chen, Rui Du

**Affiliations:** 1 College of Chinese Medicinal Materials, Jilin Agricultural University, Changchun 130118, China; 18073579409@163.com (P.G.);; 2 Jilin Provincial Engineering Research Center for Efficient Breeding and Product Development of Sika Deer, Changchun 130118, China; 3 Key Laboratory of Animal Production and Product Quality and Security, Ministry of Educatio, Ministry of National Education, Changchun 130118, China

**Keywords:** depression, 20 (S)-Protopanaxadiol, sirtuin1, dynamics-related protein 1, mitochondrial dysfunction

## Abstract

Depression is a complex and common mental illness affecting physical and psychological health. *Panax ginseng* C. A. Mey is a traditional Chinese medicine with abundant pharmacological activity and applications in regulating mood disorders. 20 (S)-Protopanaxadiol is the major intestinal metabolite of ginsenoside and one of the active components in ginseng. In this study, we aimed to investigate the therapeutic effects of 20 (S)-Protopanaxadiol on neuronal damage and depression, which may involve mitochondrial dynamics. However, the mechanism underlying the antidepressant effects of 20 (S)-Protopanaxadiol is unelucidated. In the present study, we investigated the potential mechanisms underlying the antidepressant activity of 20 (S)-Protopanaxadiol by employing a corticosterone-induced HT22 cellular model and a chronic unpredicted mild stress (CUMS)-induced animal model in combination with a network pharmacology approach. In vitro, the results showed that 20 (S)-Protopanaxadiol ameliorated the corticosterone (CORT)-induced decrease in HT22 cell viability, decrease in 5-hydroxytryptamine (5-HT) levels, and increase in nitric oxide (NO) and malondialdehyde (MDA) levels. Furthermore, 20 (S)-Protopanaxadiol exerted improvement effects on the CORT-induced increase in HT22 cell mitochondrial reactive oxygen species, loss of mitochondrial membrane potential, and apoptosis. In vivo, the results showed that 20 (S)-Protopanaxadiol ameliorated depressive symptoms and hippocampal neuronal damage in CUMS mice, and sirtuin1 (SIRT1) and peroxisome proliferator-activated receptor-1-Alpha (PGC-1α) activity were activated in the hippocampus of mice, thereby alleviating mitochondrial dysfunction and promoting the clearance of damaged mitochondria. In both in vivo and in vitro models, after inhibiting SIRT1 expression, the protective effect of 20 (S)-Protopanaxadiol on mitochondria was significantly weakened, and dynamin-related protein 1 (DRP1)-mediated mitochondrial division was significantly reduced. These findings suggest that 20 (S)-Protopanaxadiol may exert neuroprotective and antidepressant effects by attenuating DRP1-mediated mitochondrial dysfunction and apoptosis by modulating the SIRT1/PGC-1α signaling pathway.

## 1. Introduction

Depression is a heterogeneous disorder associated with persistent stress and neuronal dysfunction [1]. Depression affects the quality of life and mental and physical health of millions of people around the world [2]. Typical symptoms of depression include delayed thinking, loss of willpower, and cognitive impairment [3]. Although significant progress has been made in research on antidepressants, many antidepressants still have a series of limitations, such as severe side effects, strong drug resistance, and poor treatment effects. Previous studies have shown that individuals with depression have high suicide rates [4]. Therefore, there is an urgent need to identify new drug candidates for the treatment of depression. Thus, saponins with multiple pathways, safety, and high efficacy are the focus of antidepressant research [5].

Reportedly, traditional Chinese medicine (TCM) has a positive effect on depression, is safe, and has few adverse reactions [6,7,8,9]. Ginseng, also known as the King of Herbs, is a traditional Chinese medicine with abundant pharmacological activity. Ginseng has been used to treat mood disorders for thousands of years [9]. Our previous studies showed that ginsenosides, the main pharmacologically active components of ginseng, exert antidepressant effects. For example, ginsenoside Rg 3 can reduce cell damage caused by neurotoxicity and inhibit the elevated levels of nitric oxide (NO) and malondialdehyde (MDA) caused by glutamate [10]. Ginsenoside Rg1 prevents glial activation and dysregulation of neuronal plasticity in rats with depression [11]. 20 (S)-Protopanaxadiol, the primary ginsenoside metabolite, is an active component of ginseng and has neuroprotective properties [12]. 20 (S)-Protopanaxadiol showed strong antidepressant effects in animal models [13]. 20 (S)-Protopanaxadiol exerts antidepressant effects by activating brain-type creatine kinase in chronic corticosterone-induced rodent models [12]. However, the mechanism by which 20 (S)-Protopanaxadiol exerts its antidepressant effects remains poorly understood.

Network pharmacology is an interdisciplinary field of discovery that uses databases, high-throughput omics techniques, bioinformatics, and network visualization tools to construct multidimensional biological network models [14]. The concept of “holism” demonstrated by network pharmacology accords with the intervention mechanism of TCM with multi-component, multi-target, and multi-pathway [15]. Therefore, a combination of TCM and network pharmacology can elucidate the underlying mechanisms at the molecular level and systematically explain complex biological network relationships [16,17].

Mitochondria are important organelles that maintain cellular homeostasis. Ginsenosides help regulate mitochondrial energy metabolism, oxidative stress, biosynthesis, apoptosis, mitochondrial autophagy, and membrane channel status, and that mitochondria are important targets [18]. Compared to healthy people, there are morphological and functional changes in the mitochondria of patients with depression [19]. Abnormal changes in mitochondrial dynamics can mediate depression [20], particularly the excessive recruitment of DRP1 to the mitochondrial outer membrane, which can lead to increased mitochondrial division, resulting in abnormal function and triggering depression [21].

Hence, we used network pharmacology combined with molecular docking techniques to investigate the targets and related pathways of 20 (S)-Protopanaxadiol in the treatment of depression. On this basis, a chronic unpredictable mild stress (CUMS)-induced animal model and a corticosterone (CORT)-induced cellular model were used to investigate whether 20 (S)-Protopanaxadiol exerts its antidepressant effects through relevant targets and amelioration of mitochondrial abnormalities.

## 2. Results

### 2.1. Network Pharmacology and Molecular Docking Analysis

**Figure 1:**
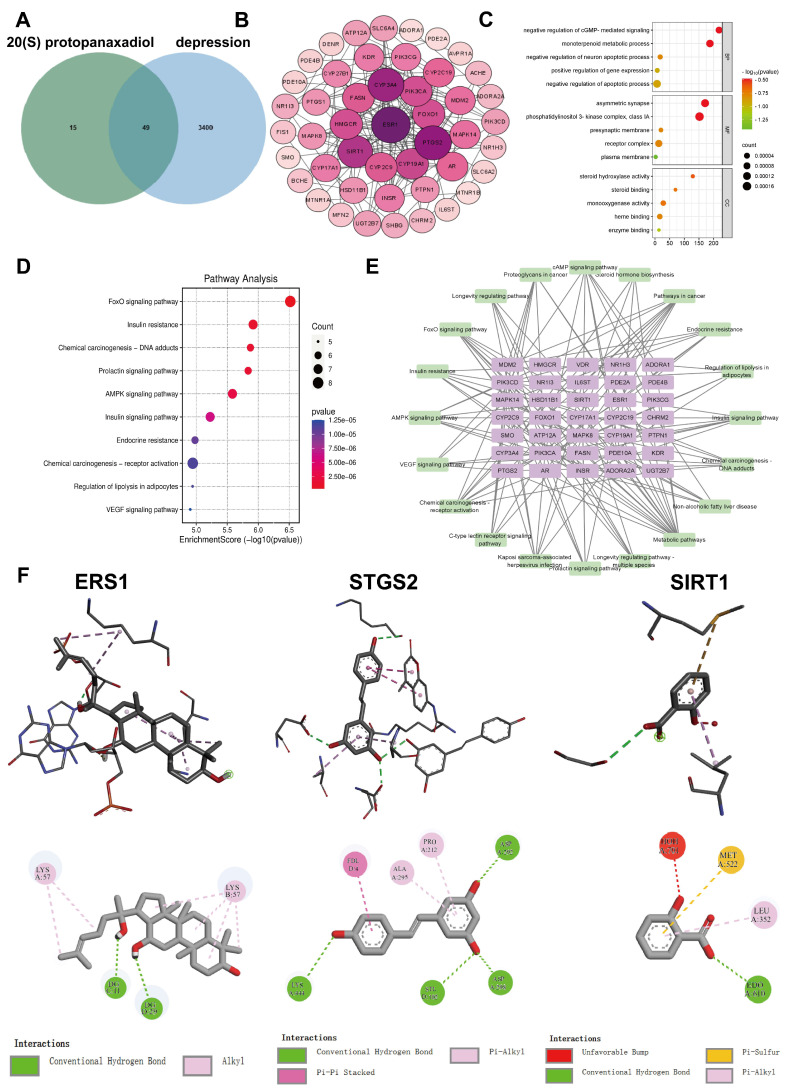
Enrichment analysis and molecular docking analysis of intersection genes between 20 (S)-Protopanaxadiol and depression. (**A**) Venn plots of Ginsenoside and depression intersection genes. (**B**) PPI network diagram of intersection genes. (**C**,**D**) Gene Ontology and Kyoto Encyclopedia of Genes and Genomes enrichment analyses. (**E**) Genes-pathway network. Purple graphics represent genes and green graphics represent pathways. (**F**) Molecular docking of 20 (S)-Protopanaxadiol to key targets (ERS1, PTGS2, and SIRT1).

The network pharmacology results showed that there were 49 intersecting genes between 20 (S)-Protopanaxadiol and depression (Figure 1A), and these 49 intersecting targets were imported into the STRING database. A PPI network diagram was drawn using Cytoscape 3.9.1, as shown in Figure 1B. GO and KEGG enrichment analyses of the intersection genes were used to explore the molecular mechanism of 20 (S)-Protopanaxadiol. The results showed that GO was enriched with a total of 191 functional relationships, including 110 biological processes (BP), 27 molecular functions (CC), and 54 cellular components (MF), mainly including positive regulation of gene expression, plasma membrane, and enzyme binding; the first five significant enrichment items of BP, CC, and MF were visualized, as shown in Figure 1C. A total of 90 pathways were enriched in KEGG. The pathways related to the antidepressant effect of 20 (S)-Protopanaxadiol mainly included the FoxO signaling pathway, chemical carcinogenesis–DNA adducts, and the AMPK signaling pathway; the first 10 important pathways were uploaded to the online platform for visual processing. Meanwhile, the intersection genes and the top 20 KEGG pathways were imported into Cytoscape 3.9.1 to draw the “genes-pathway” diagram, as shown in Figure 1E.

According to the degree value of the target, the key targets were selected for subsequent experiments using the cytoNCA plugin. The key targets were ERS1, PTGS2, and SIRT1. Molecular docking between 20 (S)-Protopanaxadiol and its key targets was performed to confirm the reliability of the target prediction results. Generally, the smaller the binding energy between the ligand and receptor, the more likely the binding is to be stable and effective. When the binding energy is ≤−5.0 kcal/mol, they are considered to have a good affinity. Molecular docking results showed that 20 (S)-Protopanaxadiol could effectively bind proteins, and its binding energies with three key targets (ERS1, STGS2, and SIRT1) were −9.9, −8.3, and −7.7 kcal/mol, respectively, all of which were <−5.0 kcal/mol. These results indicate that 20 (S)-Protopanaxadiol has good binding activity as the core target for the treatment of depression, as shown in Figure 1F.

### 2.2. Effect of 20 (S)-Protopanaxadiol on CORT-Induced HT22 Cell Viability

**Figure 2:**
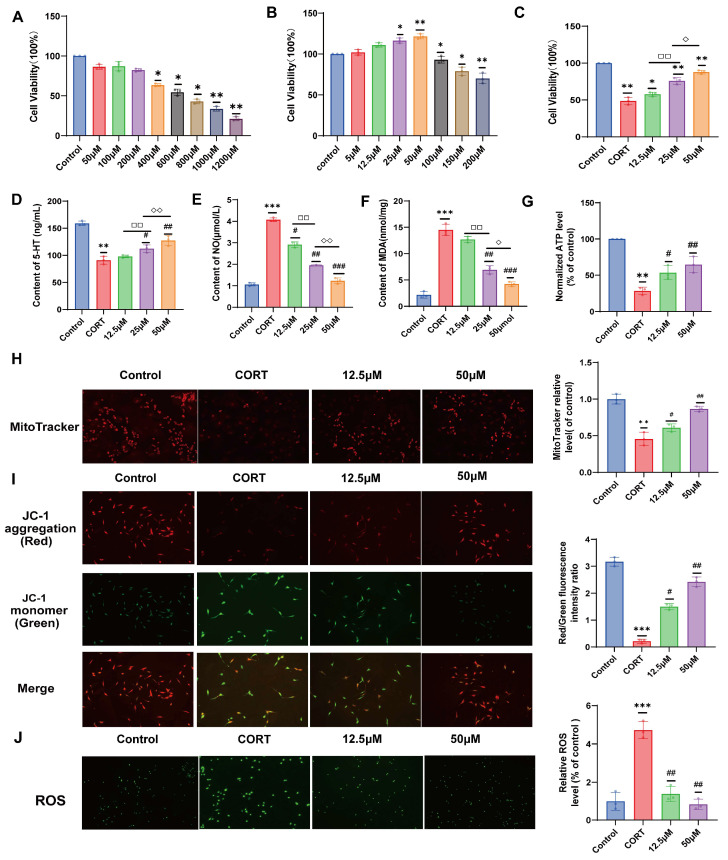
Effects of 20 (S)-Protopanaxadiol on cell viability, depression-related indicators, and mitochondrial function of HT22 cells. (**A**,**B**) Screening the optimal concentration range of 20 (S)-Protopanaxadiol and the optimal modeling concentration of CORT. (**C**) Effect of 20 (S)-Protopanaxadiol on CORT-induced HT22 cell viability. (**D**–**F**) Effects of 20 (S)-Protopanaxadiol on 5-HT, NO, and MDA contents in HT22 cells induced by CORT. (**G**) Effect of 20 (S)-Protopanaxadiol on mitochondrial ATP production in HT22 cells induced by CORT. (**H**) Effect of 20 (S)-Protopanaxadiol on mitochondrial content in HT22 cells induced by CORT. (**I**) Effect of 20 (S)-Protopanaxadiol on CORT-induced mitochondrial membrane potential in HT22 cells. (**J**) Effect of 20 (S)-Protopanaxadiol on ROS content in HT22 cells induced by CORT. All data are expressed as mean ± standard deviation. * *p* < 0.05, ** *p* < 0.01, and *** *p* < 0.001 were significantly different from the control group; ^#^ *p* < 0.05 and ^##^ *p* < 0.01 were significantly different from the CORT group; □□ *p* < 0.01 was significantly different from the 12.5 μM group; ◊ *p* < 0.05 and ◊◊ *p* < 0.01 were significantly different from the 25 μM group.

As shown in Figure 2A, cell viability was examined at CORT concentrations of 50, 100, 200, 400, 200, 800, 1000, and 1200 μM. The results showed that cortisol significantly inhibits HT22 cell proliferation in a concentration-dependent manner. When the cortisol concentration reached 600 μM, the cell survival rate was nearly 50%. Therefore, 600 μM was selected as the modeling dose of CORT. Furthermore, we selected seven different concentrations (5, 12.5, 25, 50, 100, 150, and 200 μM) of 20 (S)-Protopanaxadiol through references thesis to screen the most suitable dosing concentration range for the procedure experiment [22,23,24]. As shown in Figure 2B, compared with the CORT group, the cell survival rate gradually increased with the increase in 20 (S)-Protopanaxadiol concentration, and the cell proliferation was gradually inhibited when the concentration of 20 (S)-Protopanaxadiol reached 50 μM. In order to minimize the inhibitory effect of 20 (S)-Protopanaxadiol on cell proliferation, 12.5, 25, and 50 μM of 20 (S)-Protopanaxadiol were selected for the subsequent experiments. Finally, we examined the effect of 20 (S)-Protopanaxadiol on cell viability at 12.5, 25, and 50 μM in the presence of CORT. The results showed that HT22 cell viability increased in a dose-dependent manner and was optimal at 50 μM (Figure 2C).

### 2.3. Effects of 20 (S)-Protopanaxadiol on 5-HT, NO, and MDA Secretion in HT22 Cells Induced by CORT

As shown in Figure 2D–F, 5-HT levels were significantly decreased, and NO and MDA levels were significantly increased in the CORT group compared with those in the control group. After treatment with 20 (S)-Protopanaxadiol, the levels of 5-HT increased and the levels of NO and MDA decreased compared with those in the CORT group. Moreover, with an increase in 20 (S)-Protopanaxadiol concentration, the increase in 5-HT levels and the decrease in NO and MDA levels were more obvious. These results suggest that the ameliorative effect of 20 (S)-Protopanaxadiol on depression-related indicators was dose-dependent.

### 2.4. Effect of 20 (S)-Protopanaxadiol on Mitochondrial Content and Function of HT22 Cells Induced by CORT

ATP levels indirectly reflect the mitochondrial function. When cells are in a state of stress, apoptosis, or necrosis, the mitochondrial function is impaired, resulting in reduced ATP synthesis. As shown in Figure 2G, intracellular ATP content in HT22 cells was significantly reduced after corticosterone treatment compared with that in the control group, whereas ATP content was significantly increased after 20 (S)-Protopanaxadiol treatment compared with that in the CORT group. These results suggest that 20 (S)-Protopanaxadiol restores mitochondrial function by regulating cellular ATP levels.

MitoTracker Red CMXRos (a mitochondrial red fluorescent probe) can enter living cells directly, locate on the mitochondria, and bind covalently to them. When incubated with HT22 cells for a certain period, the mitochondria of the cells could be labeled and emitted a red fluorescence. As shown in Figure 2H, the red fluorescence of CORT group cells was weakened compared with that of control group, and the red fluorescence was enhanced after treatment with 20 (S)-Protopanaxadiol (12.5 μM, 50 μM) compared with the CORT group. These results suggest that CORT can cause mitochondrial loss, while 20 (S)-Protopanaxadiol ameliorates CORT-induced mitochondrial loss.

JC-1 is an ideal fluorescent probe that is widely used to detect mitochondrial membrane potential. When the mitochondrial membrane potential is high, JC-1 accumulates in the matrix of mitochondria under the action of mitochondrial membrane potential mainly exists in the form of J-aggregates, showing red fluorescence (Ex = 585 nm, Em = 590 nm); when the cell state is not good, the mitochondrial membrane potential decreases or loses, resulting in JC-1 mostly exists in the form of monomer in the cytoplasm, showing green fluorescence (Ex = 514 nm, Em = 529 nm). Therefore, the change in mitochondrial membrane potential can be judged according to the red–green transition and the proportional change in fluorescence color, and a decrease in mitochondrial membrane potential is an important marker of the early stage of apoptosis. As shown in Figure 2I, green fluorescence increased and red fluorescence decreased in the cells of the CORT group compared with those of the control group. After treatment with 20 (S)-Protopanaxadiol, red fluorescence increased and green fluorescence decreased compared with the CORT group. These results show that 20 (S)-Protopanaxadiol ameliorated cell damage and mitochondrial membrane potential loss.

### 2.5. Effects of 20 (S)-Protopanaxadiol on Mitochondrial ROS Activation Induced by CORT in HT22 Cells

When mitochondrial function is impaired, the excessive ROS produced by cells under external stimuli cannot be eliminated. ROS were labeled with DCFH-DA, and the resulting green fluorescence was observed under a fluorescence microscope (Figure 2J). 20 (S)-Protopanaxadiol inhibits ROS aggregation induced by cortisol.

### 2.6. Effect of 20 (S)-Protopanaxadiol on CORT-Induced Apoptosis of HT22 Cells

**Figure 3:**
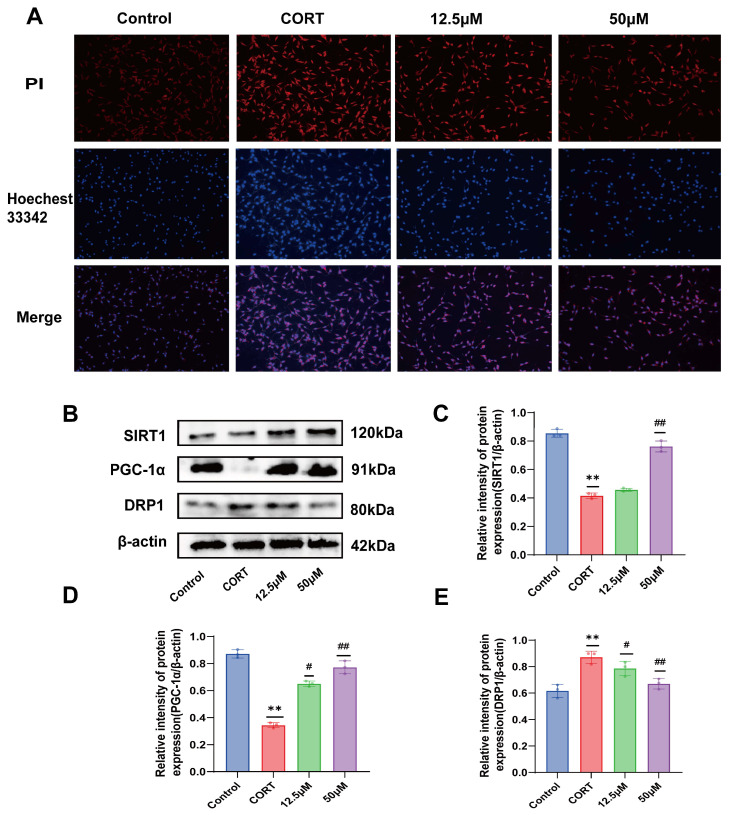
Effects of 20 (S)-Protopanaxadiol on CORT-induced apoptosis and mitochondrial dynamics-related protein expression in HT22 cells. (**A**) Hoechst 33342 /PI staining. (**B**) Expression of SIRT1, PGC-1α, and DRP1 in HT22 cells. (**C**–**E**) Results of quantitative detection of protein expression. ** *p* < 0.01 was significantly different from the control group; ^#^ *p* < 0.05 and ^##^ *p* < 0.01 were significantly different from the CORT group.

As mentioned above, a decrease in mitochondrial membrane potential is an important marker of early apoptosis; therefore, we further detected apoptosis or necrosis in HT22 cells by Hoechst 33342 and PI double staining. After staining, the cells were observed under a fluorescence microscope. Normal cells showed weak red and blue fluorescence, apoptotic cells showed weak red and strong blue fluorescence, and necrotic cells showed strong red and blue fluorescence. As shown in Figure 3A, compared with the control group, the CORT group showed significantly enhanced red and blue fluorescence, and the cells showed large areas of apoptosis and necrosis. After treatment with 20 (S)-Protopanaxadiol, the red and blue fluorescence decreased compared to that in the CORT group, indicating that 20 (S)-Protopanaxadiol could improve CORT-induced apoptosis or necrosis.

### 2.7. Effect of 20 (S)-Protopanaxadiol on CORT-Induced Mitochondrial Dynamics-Related Proteins in HT22 Cells

The protein expression levels of SIRT1, PGC-1α, and DRP1 in HT22 cells were analyzed by Western blot. As shown in Figure 3B–E, compared with the control group, SIRT1 and PGC-1α levels were significantly reduced and DRP1 level was significantly increased after CORT stimulation, and the effect of CORT on mitochondrial dynamics-related protein expression in HT22 cells was significantly improved after 20 (S)-Protopanaxadiol treatment. These results suggest that 20 (S)-Protopanaxadiol may protect HT22 cells from CORT-induced mitochondrial damage by promoting SIRT1 and PGC-1α activities and decreasing DRP1 expression.

### 2.8. Verification of the Effect of 20 (S)-Protopanaxadiol on Cell Viability and Depression-Related Indicators Using SIRT1 Inhibitor EX-527

**Figure 4:**
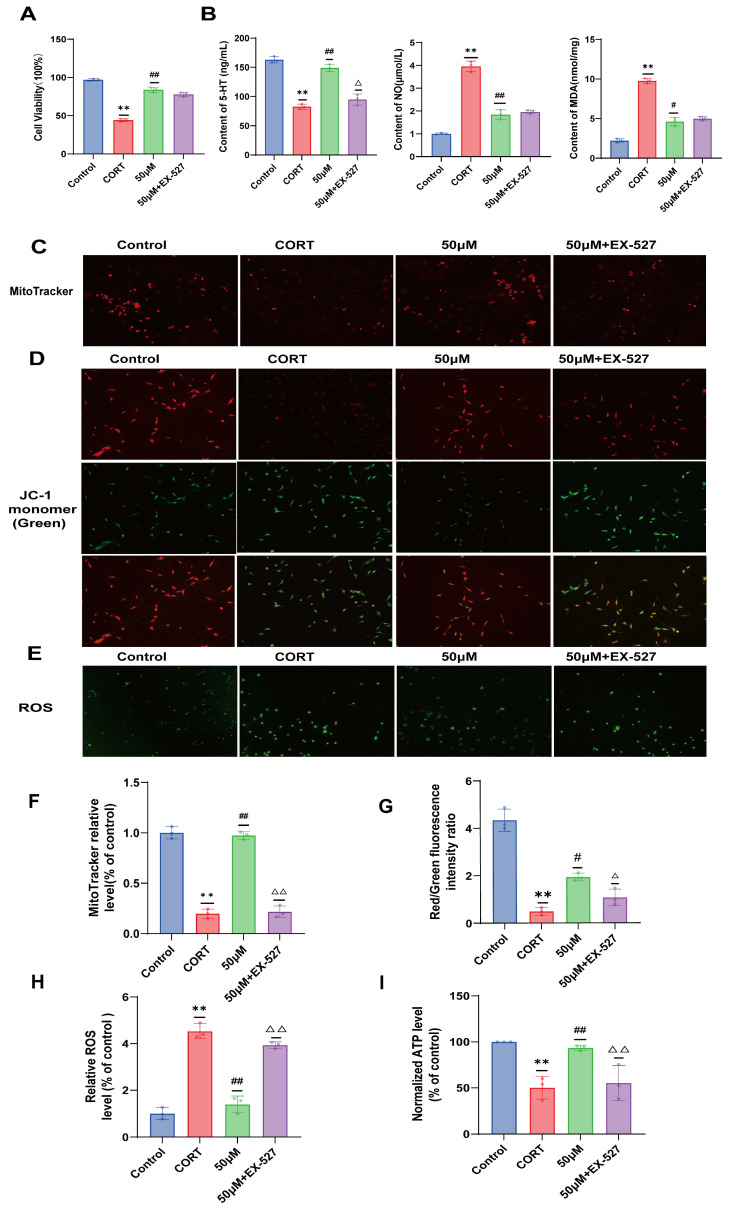
SIRT1 inhibitor EX-527 was used to examine the effects of 20 (S)-Protopanaxadiol on cell viability, depression-related indicators, mitochondrial content, and mitochondrial function. (**A**) Effect of 20 (S)-Protopanaxadiol on HT22 cell viability induced by CORT. (**B**) Effects of 20 (S)-Protopanaxadiol on 5-HT, NO, and MDA levels in HT22 cells induced by CORT. (**C**) Effect of 20 (S)-Protopanaxadiol on mitochondrial content of HT22 cells induced by CORT. (**D**) Effect of 20 (S)-Protopanaxadiol on mitochondrial membrane potential induced by CORT in HT22 cells. (**E**) Effect of 20 (S)-Protopanaxadiol on reactive oxygen species content in HT22 cells induced by CORT. (**F**–**H**) Quantitative results of mitochondrial content, mitochondrial membrane potential, and reactive oxygen species content, respectively. (**I**) Effect of 20 (S)-Protopanaxadiol on ATP content in mitochondria of HT 22 cells induced by cortisol. ** *p* < 0.01 was significantly different from control group; ^#^ *p* < 0.05 and ^##^ *p* < 0.01 significantly different from CORT group; ^△^ *p* <0.05 and ^△△^ *p* < 0.01 were significantly different from those of 20 (S)-Protopanaxadiol (50 μM) group.

As shown in Figure 4A, compared to that in the 20 (S)-Protopanaxadiol (50 μM) group, the inhibition of SIRT1 expression did not significantly decrease the viability of HT22 cells. Furthermore, as shown in Figure 4B, compared to that in the 20 (S)-Protopanaxadiol (50 μM) group, 5-HT levels in HT22 cells decreased significantly after inhibition of SIRT1 expression, whereas NO and MDA levels did not change significantly.

### 2.9. Effects of 20 (S)-Protopanaxadiol on Mitochondrial Content and Mitochondrial Function in Fine Cells Were Validated Using the SIRT1 Inhibitor EX-527

We examined the protective effect of 20 (S)-Protopanaxadiol in combination with the SIRT1 inhibitor EX-527 on CORT-induced HT22 mitochondrial content and function. The results showed that when the SIRT1 pathway was blocked, 20 (S)-Protopanaxadiol almost lost its protective effect on HT22 cell mitochondria, and there were fewer red fluorescent-labeled mitochondria in the inhibitor group than in the 20 (S)-Protopanaxadiol (50 μM) group (Figure 4C,F). The red–green fluorescence of the mitochondrial membrane potential was similar to that of the model group (Figure 4D,G). ROS accumulation again increased (Figure 4E,H) and mitochondrial ATP production again decreased (Figure 4I) compared with the 20 (S)-Protopanaxadiol (50 μM) group. 20 (S)-Protopanaxadiol inhibited CORT-induced mitochondrial damage through the SIRT1 pathway.

### 2.10. Verification of the Effect of 20 (S)-Protopanaxadiol on Apoptosis Using SIRT1 Inhibitor EX-527

**Figure 5:**
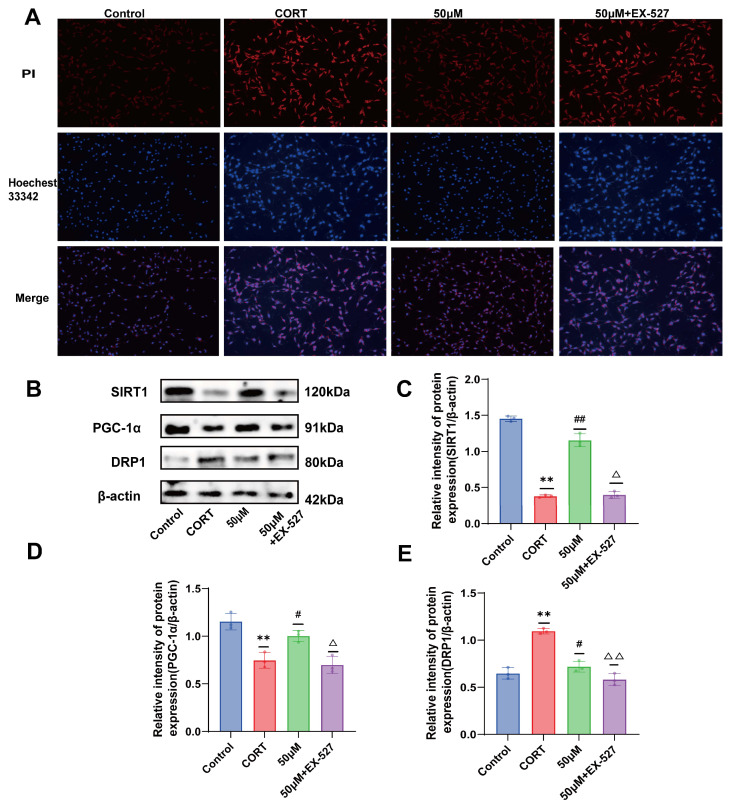
SIRT1 inhibitor EX-527 was used to examine the effects of 20 (S)-Protopanaxadiol on apoptosis and mitochondrial dynamics-related proteins. (**A**) Hoechst 33342 /PI staining. (**B**) Expression of SIRT1, PGC-1α, and DRP1 in HT22 cells. (**C**–**E**) Quantitative detection results of protein expression. ** *p* < 0.01 was significantly different from control group; ^#^ *p* <0.05 and ^##^ *p* < 0.01 significantly different from CORT group; ^△^ *p* < 0.05 and ^△△^ *p* < 0.01 were significantly different from those of 20 (S)-Protopanaxadiol (50 μM) group.

The results showed that when SIRT1 expression was inhibited, the red and blue fluorescence of HT22 cells in the inhibitor group was increased, and a large number of cells showed apoptosis and necrosis, compared with that in the 20 (S)-Protopanaxadiol (50 μM) group (Figure 5A). These results suggest that 20 (S)-Protopanaxadiol inhibits CORT-induced apoptosis and necrosis of HT22 cells via the SIRT1 pathway.

### 2.11. Validation of the Effect of 20 (S)-Protopanaxadiol on Mitochondrial Dynamics-Related Proteins Using SIRT1 Inhibitor EX-527

Western blot results showed that after the application of EX-527, the expressions of SIRT1 and PGC-1α were significantly decreased, while the expression of DRP1 was significantly increased, compared with 20 (S)-Protopanaxadiol (50 μM) group (Figure 5B). The quantitative results are shown in Figure 5C–E. These results suggest that 20 (S)-Protopanaxadiol may inhibit drp1-mediated mitochondrial division by participating in the SIRT1 activity pathway.

### 2.12. Behavioral Results

**Figure 6:**
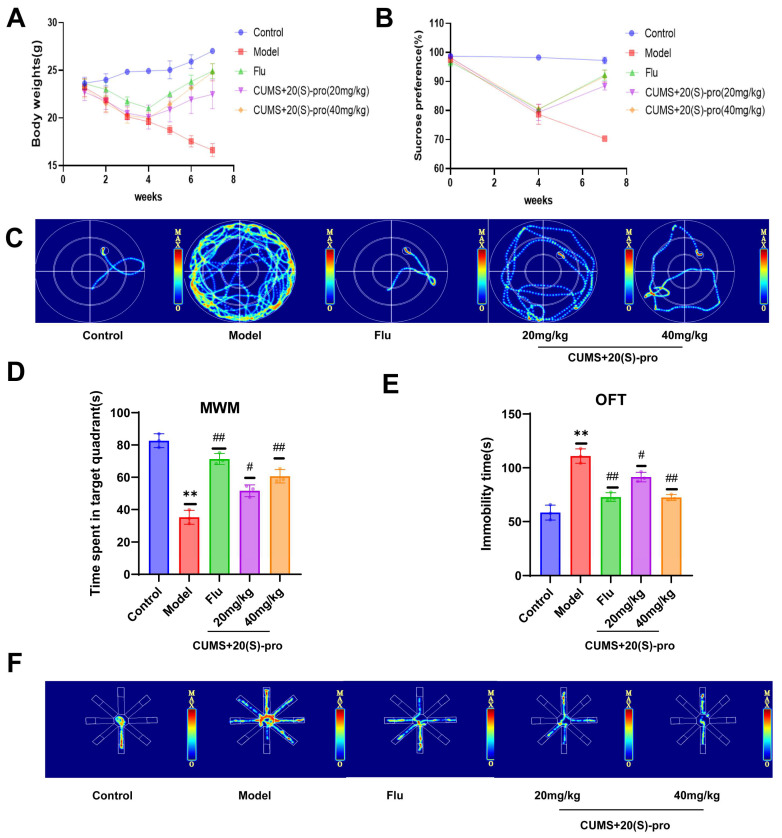
Effects of 20 (S)-Protopanaxadiol (labeled as 20 (S)-pro in the figure) on depression-like behavior. (**A**) Changes in body weight of mice in each group over 7 weeks. (**B**) Changes in sucrose preference rate of mice in each group within 7 weeks. (**C**) MWM mouse activity thermogram. (**D**) Dwell time of mice in MWM target quadrant. (**E**) Immobility time of mice in OFT. (**F**) Activity heat map of 8-arm maze mice. All data are expressed as mean ± standard deviation, *n* = 8. ** *p* < 0.01 was significantly different from the control group; ^#^ *p* < 0.05 and ^##^ *p* < 0.01 were significantly different from CUMS group.

Throughout the experiment, the body weight of the mice in the control group increased steadily. Compared with the control group, the CUMS model group showed a significant reduction in body weight. After the administration of Flu and 20 (S)-Protopanaxadiol, the situation improved significantly compared to that in the model group (Figure 6A). Second, the antidepressant effect of 20 (S)-Protopanaxadiol was evaluated using 4 behavioral manifestations: SPT, MWM, OFT, and the 8-Arm Maze. As shown in Figure 6B, the sucrose preference index of the CUMS model mice decreased significantly compared with that of the control group, and the sucrose preference rate of the Flu and 20 (S)-Protopanaxadiol) group mice increased in a dose-dependent manner after drug treatment compared with that of the model group. The MWM results showed that the model mice spent less time in the target quadrant compared to the control group and that the time spent in the target quadrant was significantly increased after the use of Flu and 20 (S)-Protopanaxadiol compared to the model group (Figure 6C,D). OFT results showed that the immobility time of mice in CUMS group was significantly longer than that in control group. In contrast, treatment with Flu and 20 (S)-Protopanaxadiol significantly reduced the immobility time in OFT in mice compared to the model group (Figure 6E). An 8-Arm maze was used to test learning and memory performance in a brain-damaged state. As shown in Figure 6F, mice in the CUMS group stayed significantly longer in the non-feeding arm than in the control group, whereas mice in the 20 (S)-Protopanaxadiol and Flu groups stayed significantly shorter in the non-feeding arm than in the model group. The above results showed that 20 (S)-Protopanaxadiol improved the spatial memory and learning ability, activity ability, and exploration ability of mice.

### 2.13. Expression of 5-HT, MDA, and ATP in Serum

**Figure 7:**
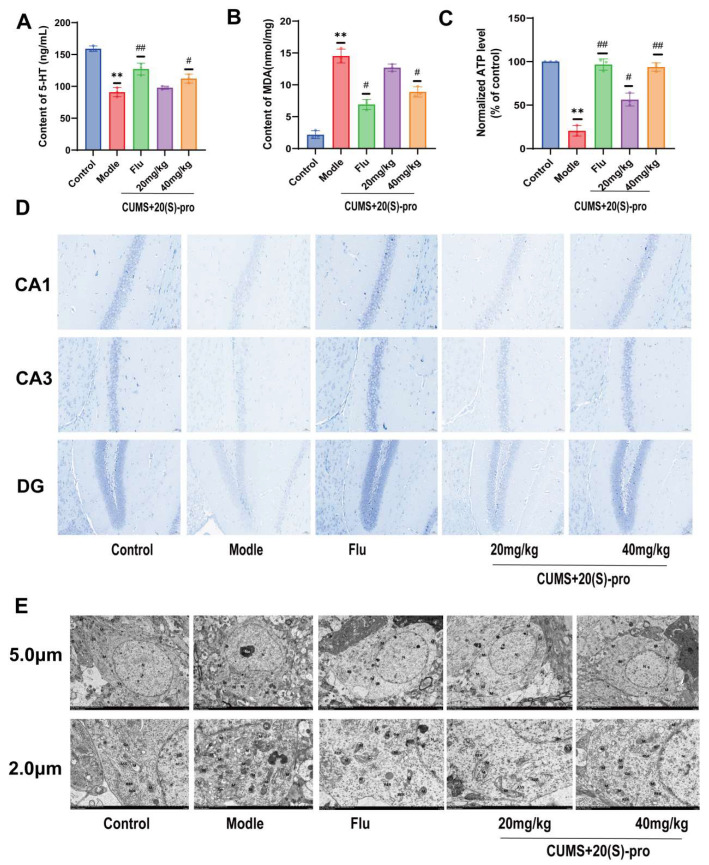
Effects of 20 (S)-Protopanaxadiol (labeled as 20 (S)-pro in the figure) on serum depression-related factors, hippocampus neurons, hippocampus mitochondria morphology, and mitochondria function in mice. (**A**,**B**) Determination of serum 5-HT, MDA content. (**C**) Determination of ATP content in the hippocampus. (**D**) Nissl staining results image of the hippocampus. (**E**) Electron microscope image of hippocampus tissue. All data are expressed as mean ± standard deviation. ** *p* < 0.01 was significantly different from the control group; ^#^ *p* < 0.05 and ^##^ *p* < 0.01 were significantly different from the model group.

The levels of 5-hydroxytryptamine (5-HT) have been linked to depression and the effectiveness of antidepressants. Figure 7A shows that 5-HT levels in mouse serum decreased after CUMS treatment compared to the control group and significantly increased after Flu 20 (S)-Protopanaxadiol treatment compared to the model group. The MDA is commonly used to detect depression in clinical practice. As shown in Figure 7B, MDA expression levels in the serum of mice were significantly increased after CUMS treatment compared to the control group and significantly decreased after Flu 20 (S)-Protopanaxadiol treatment compared to the model group. These results suggest that 20 (S)-Protopanaxadiol may exert its antidepressant effects by increasing the expression of 5-HT and inhibiting MDA expression. Furthermore, because ATP levels indirectly reflect mitochondrial function, which is closely related to the onset of depression, we measured ATP levels in the hippocampi of mice. As shown in Figure 7C, ATP levels decreased significantly after CUMS treatment compared to those in the control group and increased dramatically after Flu 20 (S)-Protopanaxadiol treatment compared to those in the model group.

### 2.14. Effect of 20 (S)-Protopanaxadiol on CUMS-Induced Hippocampal Neuronal Function in Mice

To investigate the protective effect of 20 (S)-Protopanaxadiol against hippocampal neuronal injury in mice, morphological and quantitative changes in neurons and cell bodies in the CA1, CA3, and DG regions of the hippocampus were observed using the Nissl staining method. Nissl staining showed that compared with the control group, the neurons in the hippocampus of the model group mice were sparsely arranged, with abnormal cell morphology, decreased neuron density, reduced volume of most neurons, and decreased number of Nissl bodies. Compared with those in the model group, the pathological morphology of hippocampal neurons in the 20 (S)-Protopanaxadiol and Flu groups was significantly improved; neurons were arranged neatly and densely, and Nissl bodies were visible (Figure 7D). The results showed that 20 (S)-Protopanaxadiol could significantly improve the damage of hippocampal neurons induced by depression in mice.

### 2.15. Effect of 20 (S)-Protopanaxadiol on Mitochondrial Morphology and Related Functions in the Hippocampus of Mice

The ultrastructure of hippocampal mitochondria was observed using transmission electron microscopy, as shown in Figure 7E. In the model group, most of the hippocampal mitochondria (M) were significantly swollen and enlarged, the membrane was damaged, the matrix was dissolved and overflowed, and the cristae were broken and dissolved compared to those in the control group. After Flu and 20 (S)-Protopanaxadiol administration, the degree of mitochondrial swelling (M) was significantly reduced, the size was uniform, the membrane was relatively intact, the matrix was shallow, and the incidence of ridge breakage and shortening was reduced compared with that in the model group. The above results indicated that 20 (S)-Protopanaxadiol had an ameliorating effect on the damage of mitochondrial morphology and function in the mouse hippocampus caused by CUMS.

### 2.16. Effect of 20 (S)-Protopanaxadiol on Mitochondrial Dynamics-Related Protein Expression

**Figure 8:**
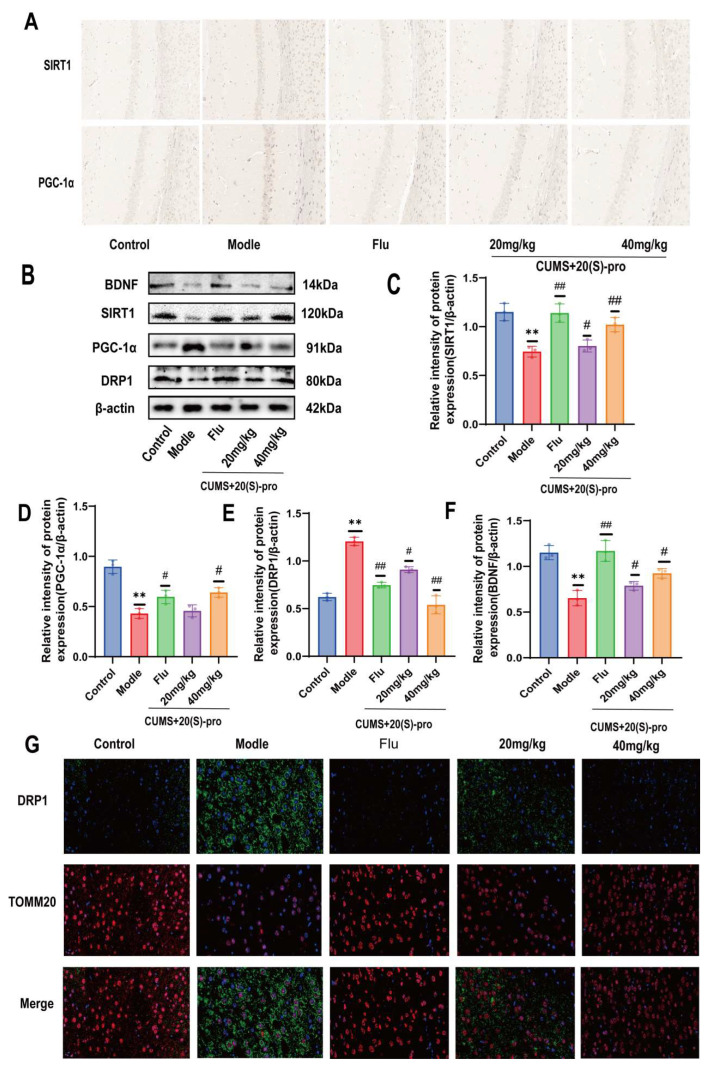
Effect of 20 (S)-Protopanaxadiol (labeled as 20 (S)-pro in the figure) on mitochondrial dynamics-related protein expression. (**A**) Immunohistochemical signal intensity of SIRT1 and PGC-1α in hippocampus. (**B**) Expression of SIRT1, PGC-1α, DRP1, and BDNF in hippocampus. (**C**–**F**) Results of quantitative detection of protein expression in sea. (**G**) Immunofluorescence images show the colocalization of DRP1 (green) and TOMM20 (red). The nuclei were stained with 2-(4-amidinophenyl)-6-indolecarbamidine dihydrochloride (DAPI). All data are expressed as mean ± standard deviation, *n* = 8. ** *p* < 0.01 was significantly different from the control group; ^#^ *p* < 0.05 and ^##^ *p* < 0.01 were significantly different from the model group.

SIRT1 is a key target at the intersection of 20 (S)-Protopanaxadiol and depression and has good docking activity in molecular docking validation. Furthermore, SIRT1 typically increases mitochondrial biosynthesis by activating the deacetylated peroxisome proliferator-activated receptor-γ-coactivator 1α (PGC-1α). To determine the molecular mechanisms underlying mitochondrial morphological damage and fragmentation, we investigated the expression levels of a protein involved in mitochondrial division (DRP1). We first detected the expression of SIRT1 and PGC-1α protein in the hippocampus of mice by immunohistochemistry. The density of SIRT1 and PGC-1α positive microglia in the hippocampus of mice in the model group decreased significantly compared with the control group. The density of SIRT1 and PGC-1α positive microglia increased dramatically after Flu and 20 (S)-Protopanaxadiol treatment compared with the model group (Figure 8A). Then, the expression levels of SIRT-1, PGC-1α, and DRP1 in the hippocampus were detected by Western blotting, and the results also showed that the expression levels of SIRT1 and PGC-1α in the hippocampus of the model group were decreased, while the expression level of DRP1 was increased compared with the control group. And compared with the model group, the expression levels of SIRT1 and PGC-1α increased significantly after Flu and 20 (S)-Protopanaxadiol treatment, while the expression levels of DRP1 decreased (Figure 8B). The quantitative results are shown in Figure 8C–E. As BDNF is also a common marker of depression, we examined BDNF expression levels in the hippocampi of mice. BDNF levels in the hippocampus of mice were significantly reduced after CUMS treatment compared to those in the control group, whereas BDNF levels increased again after Flu and 20 (S)-Protopanaxadiol administration compared to those in the model group, as shown in Figure 8B,F.

Finally, to verify the effect of DRP1 on mitochondrial division, dual immunofluorescence staining of DRP1 (green) and translocase of the outer mitochondrial membrane (TOMM20) (red) in hippocampal nerve cells was performed (Figure 8G). The results showed that the accumulation of DRP1 in the outer mitochondrial membrane increased after CUMS treatment. Pooled images showed that DRP1 and mitochondrial co-localization were more abundant in the model group than in the control group. After treatment with Flu and 20 (S)-Protopanaxadiol, the co-localization of DRP1 with mitochondria was significantly weakened compared to that in the model group. These results suggest that 20 (S)-Protopanaxadiol exerts its antidepressant effects by promoting SIRT1 activity, decreasing DRP1 expression, and inhibiting DRP1 aggregation in the outer mitochondrial membrane.

### 2.17. SIRT1 Inhibitor EX-527 Verified the Effect of 20 (S)-Protopanaxadiol on Mitochondrial Ultrastructure and Function

**Figure 9:**
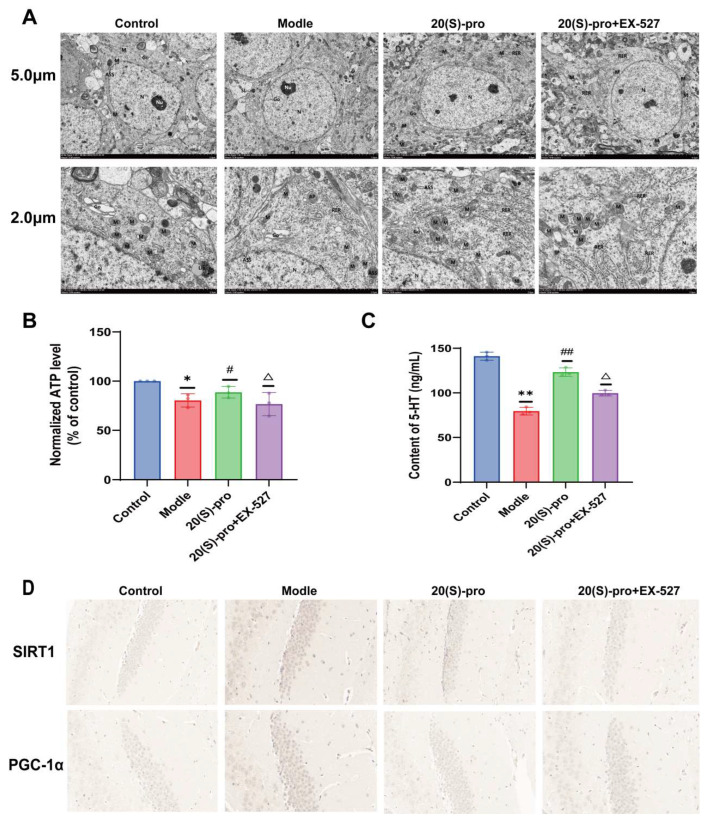
To verify the effects of 20 (S)-Protopanaxadiol (labeled as 20 (S)-pro in the figure) on mitochondrial ultrastructure and function. (**A**) Electron microscope image of hippocampus tissue. (**B**) Determination of ATP content in the hippocampus. (**C**) Determination of serum 5-HT content. (**D**) Immunohistochemical signal intensity of SIRT1 and PGC-1α in hippocampus. All data are expressed as mean ± standard deviation. * *p* < 0.05 and ** *p* < 0.01 were significantly different from the control group; ^#^ *p* < 0.05 and ^##^ *p* < 0.01 were significantly different from the model group; and ^△^ *p* < 0.05 was significantly different from the 20 (S)-pro group.

We investigate whether 20 (S)-Protopanaxadiol could improve the morphology and function of mitochondria in the hippocampus of mice by regulating the expression of SIRT1 and downstream-related proteins and exerting antidepressant effects. We used a selective inhibitor of SIRT1 (EX-527) to inhibit SIRT1 expression in the mouse hippocampus. Consistent with previous results, most of the hippocampal mitochondria (M) in the model group mice were swollen, membrane-damaged, matrix-lysed, and ridges shortened and ruptured in comparison with the control group. After Flu and 20 (S)-Protopanaxadiol administration, mitochondrial damage significantly improved in comparison with that in the model group. After the application of EX-527, most of the hippocampal mitochondria were still in a damaged state compared to the 20 (S)-Protopanaxadiol (40 mg/kg) group (Figure 9A). Compared with the control group, the ATP level in the hippocampus of mice in the model group was significantly reduced, and the ATP level was significantly increased after Flu and 20 (S)-Protopanaxadiol treatment compared with the model group; the ATP level decreased again after EX-527 administration in comparison with the 20 (S)-Protopanaxadiol (40 mg/kg) group (Figure 9B). Furthermore, we examined serum 5-HT levels in mice and found that the inhibition of SIRT1 expression inhibited the promotion of 5-HT expression by 20 (S)-Protopanaxadiol (Figure 9C). These results suggest that 20 (S)-Protopanaxadiol has a protective effect on CUMS-induced mitochondrial damage, and this protective effect may depend on SIRT1 activity.

### 2.18. SIRT1 Inhibitor EX-527 Verified the Effect of 20 (S)-Protopanaxadiol on the Expression of Mitochondrial Dynamics-Related Proteins

**Figure 10:**
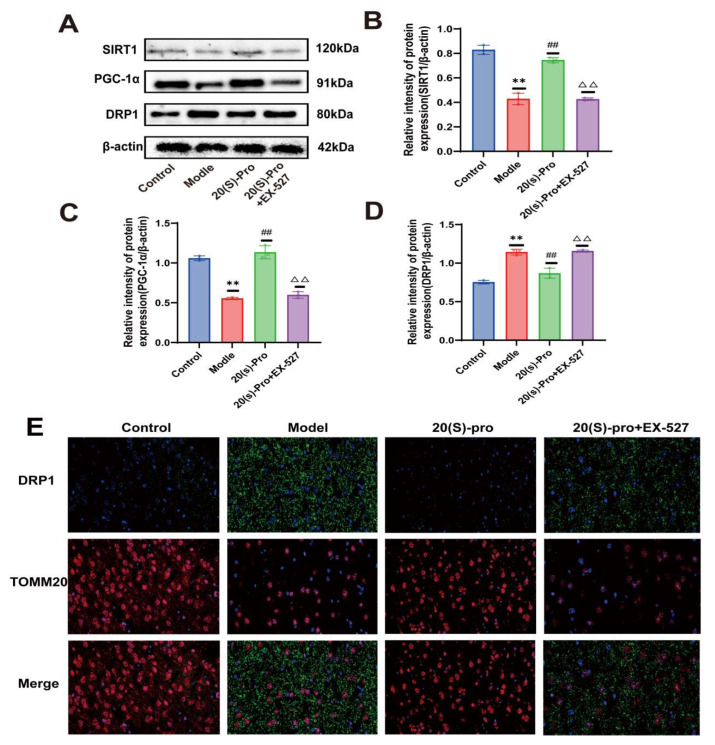
To verify the effect of 20 (S)-Protopanaxadiol (labeled as 20 (S)-pro in the figure) on mitochondrial dynamics-related protein expression. (**A**) Expression of SIRT1, PGC-1α, and DRP1 in hippocampus. (**B**–**D**) Results of quantitative detection of protein expression in sea. (**E**) Immunofluorescence images show the colocalization of DRP1 (green) and TOMM20 (red). All data are expressed as mean ± standard deviation. ** *p* < 0.01 was significantly different from the control group; ^##^ *p* < 0.01 was significantly different from the model group; ^△△^ *p* < 0.01 was significantly different from the 20 (S)-pro group.

Consistent with the previous results, the density of SIRT1- and PGC-1α-positive microglia in the hippocampus of mice in the model group was significantly reduced compared with the control group, and the condition was improved after Flu and 20 (S)-Protopanaxadiol treatment compared to the model group. After the application of EX-527, the density of SIRT1- and PGC-1α-positive microglia decreased again in comparison with the 20 (S)-Protopanaxadiol (40 mg/kg) group (Figure 9D). Furthermore, compared to the control group, the results of Western blotting also showed that the expression levels of SIRT1 and PGC-1α in the hippocampus of the model group were decreased, while the expression level of DRP1 was increased. Compared to the model group, the expression levels of SIRT1 and PGC-1α increased significantly after Flu and 20 (S)-Protopanaxadiol treatment, while the expression level of DRP1 decreased. After the application of EX-527, the expression of SIRT1 and PGC-1α again decreased and the expression of DRP1 again increased compared with the 20 (S)-Protopanaxadiol (40 mg/kg) group (Figure 10A). The quantitative results are shown in Figure 10B–D.

Finally, dual immunofluorescence staining of DRP1 (green) and TOMM20 (red) is also shown (Figure 10E); DRP1 and mitochondrial colocalization became evident again after the application of EX-527 in comparison with the 20 (S)-Protopanaxadiol (40 mg/kg) group. The results showed that 20 (S)-Protopanaxadiol inhibited DRP1 aggregation into mitochondrial outer membrane and restored the expression of SIRT1 pathway. After inhibition of SIRT1 expression, DRP1 accumulation to mitochondrial outer membrane resumed. These results suggest that 20 (S)-Protopanaxadiol inhibits DRP1-mediated mitochondrial division by participating in SIRT1 activity.

## 3. Materials and Methods

### 3.1. Materials

20 (S)-Protopanaxadiol, Fluoxetine hydrochloride, Corticosterone (Yuanye Biotechnology, Shanghai, China); Selisistat (EX-527, an sirtuin1 (SIRT1) inhibitor, MedchemExpress, Shanghai, China); Mouse 5-HT ELISA kit (Ruixin Biotechnology, Quanzhou, China); Malondialdehyde (MDA) assay kit (BYabscience, Nanjing, China); Total Nitric Oxide (NO) Assay Kit (Beyotime Biotechnology, Shanghai, China); Bicinchoninic acid assay (BCA) protein assay kit, phenylmethylsulfonyl fluoride (PMSF), radio immunoprecipitation assay (RIPA) Lysis Buffer, 5 × SDS-PAGE protein loading buffer phosphate buffer saline (PBS) buffer solution, Tris-buffered saline with Tween^®^ 20 (TBST) solution, tris-glycine SDS-PAGE electrophoresis buffer, and transmembrane buffer (Servicebio Biotechnology, Wuhan, China); polyvinylidene fluoride (PVDF) membranes (0.45 μm, Millipore, Darmstadt, Germany); Anti-SIRT1 (1:1500); Anti-PGC-1α (1:1500), Anti-DRP1 (1:1500), Anti-β-actin (1;1000), Anti-BDNF (1:800) (Wanleibio, Shenyang, China); HRP Goat Anti-Rabbit IgG (1:3000, Servicebio, Wuhan, China); ECL Chemiluminescence Kit (biosharp, Hefei, China); DMEM culture medium (Gibco, Beijing, China); PBS and premium fetal bovine serum (FBS) (Clark Bioscience, Richmond, VA, America); MitoTracker Red CMXRos, Reactive Oxygen Species Assay (ROS) Kit and JC-1 Mitochondrial Membrane Potential Assay Kit (Servicebio Biotechnology, Wuhan, China); ATP content determination kit (Aidisheng Biotechnology, Shanghai, China) were used in this study.

### 3.2. Target Acquisition of 20 (S)-Protopanaxadiol and Depression

Traditional Chinese Medicine Systems Pharmacology (TCMSP, https://old.tcmsp-e.com/tcmsp.php, accessed on 3 February 2023), PubChem (https://pubchem.ncbi.nlm.nih.gov/, accessed on 7 February 2023), and the Swiss Target Prediction Database (STP, http://www.swisstargetprediction.ch/, accessed on 10 February 2024) were used to obtain the targets for 20 (S)-Protopanaxadiol. The UniProt database (https://www.uniprot.org/, accessed on 14 February 2023), Genecards database (https://www.genecards.org/, accessed on 16 February 2023), and online Human Mendelian Gene Database (OMIM, https://omim.org/, accessed on 19 February 2023) were used to collect targets for depression using the keyword “depression”.

### 3.3. Construction of the PPI Network

The obtained 20 (S)-Protopanaxadiol and depression targets were uploaded to the online platform of Weishengxin (http://www.bioinformatics.com.cn/, accessed on 23 February 2023) for Venn mapping, and intersection genes were obtained. The cross genes were then imported into the STRING (https://string-db.org/, accessed on 26 February 2023) database for protein interaction analysis, and the results were imported into Cytoscape 3.9.1 software to make a protein–protein interaction (PPI) network diagram. The top 10 genes were visualized using the cytoNCA plug-in as a basis for target gene selection in subsequent experiments [25].

### 3.4. GO Functional Enrichment Analysis and KEGG Pathway Enrichment Analysis

Genes were intersected into the David (https://david.ncifcrf.gov/, accessed on 27 February 2023) database for gene ontology (GO) and Kyoto Encyclopedia of Genes and Genomes (KEGG) enrichment analysis. The top 10 results of KEGG enrichment analysis and the top 5 results of biological process (bioprocess, BP), molecular function (MF), and cell component (CC) in GO analysis were uploaded to the WeiShengxin platform for visualization.

### 3.5. Construction of the “Genes-Pathway” Network Map

According to the top 20 KEGG signaling pathways selected by the David database and the intersection genes obtained in the above steps, they were sorted into network files and type files and imported into Cytoscape 3.9.1 software to build a “genes-pathway” network map.

### 3.6. Molecular Docking

Three core targets were selected for molecular docking validation with 20 (S)-Protopanaxadiol. The three-dimensional structure of the protein encoded by the core target gene was downloaded from a protein database (https://www.rcsb.org/, accessed on 5 March 2023). The 2D structure of 20 (S)-Protopanaxadiol was downloaded from the PubChem database (https://pubchem.ncbi.nlm.nih.gov/, accessed on 8 March 2023) and saved in “mol2” format. Chemoffice 2020 software (PerkinElmer Informatics, Shelton, CT, USA) was used to convert the two-dimensional structure of 20 (S)-Protopanaxadiol to a three-dimensional structure, and AutoDock Tools 1.5.6 software was used for hydrogenation, structure optimization, and output generation in the “pdbqt” format of the ligand. Active docking pockets were created using the GetBox plug-in of Pymol 2.3 software (DeLano Scientific LLC, South San Francisco, CA, USA), and the spatial location and radius of the active docking pockets were recorded. Molecular docking of the receptor and ligand was performed using AutoDock Vina 1.1.2 software, and binding energies were calculated using Vina scripts. The results were visualized using Pymol 2.3 software.

### 3.7. Screening of Optimal CORT Molding Concentration and Optimal Administration of 20 (S)-Protopanaxadiol

HT22 cells were added to 96-well plates at a concentration of 5 × 10^4^ cells/mL and incubated at 37 °C. First, 50, 100, 200, 400, 200, 800, 1000, and 1200 μM of CORT [26] were added to each well, and the cell viability of each well was detected by the CCK-8 kit (Biosharp, Hefei, China). The optimal CORT concentration for cell damage was selected when cell viability was close to 50%. Meanwhile, each well was treated with 100 μL of 20 (S)-Protopanaxadiol solution at different concentrations (5, 12.5, 25, 50, 100, 150, and 200 μM) for 24 h. The safe concentration of 20 (S)-Protopanaxadiol was determined based on cell viability. Finally, under the conditions of CORT modeling, the safe concentration of 20 (S)-Protopanaxadiol was tested using the CCK-8 kit to determine whether the promotional effect of 20 (S)-Protopanaxadiol on HT22 cell proliferation was dose-dependent. If so, two of these concentrations were selected for subsequent experiments.

### 3.8. Cell Modeling, Grouping, and Administration

HT22 cells were cultured in Dulbecco’s modified eagle’s medium (DMEM) containing 10% heat-inactivated FBS, 1% penicillin, and streptomycin and subcultured in a cell incubator at 37 °C and 5% CO_2_. Cells were divided into the control group, CORT group, CORT + 20 (S)-Protopanaxadiol (12.5 μM) group, and CORT + 20 (S)-Protopanaxadiol (50 μM) group. To verify whether 20 (S)-Protopanaxadiol exerts mitochondrial protection on HT22 cells via SIRT1/PGC-1α, we added an experimental group (CORT + 20 (S)-Protopanaxadiol (50 μM)+EX-527 (10 μM)) [27]. The model cells were cultured in a medium containing CORT (600 μM) for 24 h to establish a neuronal injury model, and various drugs were administered 24 h after modeling. The control group did not receive drug stimulation or treatment. Cell viability of each group (including the control group) was assessed using the CCK-8 kit; 10 μL CCK-8 solution was added to each well, cultured in a 37 °C incubator for 2 h, and measured at 450 nm using a microplate reader (Epoch2; Burton Instruments Co., LTD, Gainesville, FL, USA).

### 3.9. Determination of 5-HT, NO, and MDA Expression in HT22 Cells

After the incubation of cells and drugs, the concentrations of 5-HT, NO, and MDA in the cell culture medium were estimated using an enzyme marker, and the absorbance was read at 450, 540, and 450 nm according to the guiding principles of the ELISA kit and the method provided by the manufacturer.

### 3.10. ATP Content Detection in Hippocampus and Cells

An ATP content detection kit was used to detect the ATP content in the hippocampal tissues and cells. Hippocampal tissue and drug-treated cells were added to the homogenate and centrifuged at 12,000 rpm for 10 min at room temperature. The supernatant was collected for later use. The supernatant was mixed with the reaction solution according to the manufacturer’s instructions, and the absorbance value of each tube was read at 700 nm in a water bath at 37 °C for 20 min. Finally, the ATP content of each sample was calculated according to the manufacturer’s instructions.

### 3.11. Determination of Mitochondrial Membrane Potential and ROS Production

The JC-1 mitochondrial membrane potential detection kit and ROS detection kit were used to detect mitochondrial membrane potential and ROS production in HT22 cells, respectively. According to the kit’s package insert, after drug treatment, the cells are taken, the medium is removed, the reaction solution is added, and the cells are incubated at 37 °C for 30 min in the dark. The cells were observed under a fluorescence microscope.

### 3.12. MitoTracker Red CMXRos Labeling Mitochondria

According to the method provided by the kit manufacturer, the mitochondria were labeled with a red fluorescent probe, incubated in an incubator at 37 °C for 30 min, removed the incubation fluid, cleaned with buffer solution 2–3 times for 3–5 min each time, sealed with anti-fluorescence quenching sealing solution, and observed under a fluorescence microscope.

### 3.13. Apoptosis Assays

Apoptosis was detected by Hoechst 33342 and propidium iodide (PI) double staining. The cells (1 × 10^6^ cells/well) were seeded onto coverslips in 12-well plates. The cells were treated with different drugs and incubated in a carbon dioxide incubator at 37 °C for 24 h. After incubation, fresh culture medium was added, and 5 µL Hoechst staining solution and 5 µL PI staining solution were added to each well; mixed well; incubated at 4 °C for 20–30 min; and observed red and blue fluorescence under a laser confocal microscope.

### 3.14. Experimental Animals and Study Design

Six-week-old male C57BL/6 mice (22–25 g) were obtained from Changchun Yisi Laboratory Animal Technology Co., Ltd. (Changchun, China). Experimental animals were housed at the Laboratory Animal Research Center of Jilin Agricultural University. The animals were fed under standard conditions (20–22 °C), with light and dark cycles for 12 h, and food and water were freely ingested. The animals were adaptively fed for 1 week before the experiment. The experiment was approved by the Animal Care Professional Committee of Jilin Agricultural University, ethical review acceptance number: 2021 10 11 003.

The CUMS model was created with minor changes from the reference literature, including fasting, no water, day and night reversal, the 45-degree tilt of the cage, shaking of the cage, wet bedding, noise stimulation, ice water swimming, and placement of foreign objects in the cage [28]. The mice were subjected to random CUMS stimulation every day for seven consecutive weeks, and their body weights were recorded weekly.

Experiment 1: Effect of 20 (S)-Protopanaxadiol on CUMS-induced Depression Model Mice C57/BL6 mice were randomly divided into five groups (*n* = 8): control, CUMS, CUMS + fluoxetine (10 mg/kg) [29,30] group (CUMS + FLU), CUMS + 20 (S)-Protopanaxadiol (20 mg/kg) [31,32,33,34] group, and CUMS + 20 (S)-Protopanaxadiol (40 mg/kg) [31,33,34]. Oral administration of 20 (S)-Protopanaxadiol and FLU for 3 weeks, starting at week 5 of CUMS. Behavioral testing was performed 24 h after the final dose. Subsequently, mice were sacrificed, some hippocampal tissue was preserved at 80 °C, and some hippocampal tissue was soaked in paraformaldehyde fixative or electron microscope fixative at 4 °C for further testing.

Experiment 2: Effect of SIRT1 Inhibitor EX-527 on the Action of 20 (S)-Protopanaxadiol C57/BL6 mice were randomly divided into four groups (*n* = 8): control group, CUMS group, CUMS + 20 (S)-Protopanaxadiol (40 mg/kg) group (CUMS + 20 (S)-Protopanaxadiol), and CUMS+20 (S)-Protopanaxadiol (40 mg/kg) + EX-527 (5 mg/kg) [35] group (CUMS + 20 (S)-Protopanaxadiol +EX-527). Starting from the 5th week of CUMS modeling, 20 (S)-Protopanaxadiol (40 mg/kg) was administered by gavage for 3 weeks, and EX-527 (5 mg/kg) was injected intraperitoneally from the 6th week of CUMS modeling for 2 weeks. Subsequent experiments were conducted, including those used in Experiment 1.

### 3.15. Sucrose Preference Test (SPT)

Sugar water (usually a 1% sucrose solution) was given to the animals 1–2 days before the SPT, to acclimate to the taste of sugar water. On the day of the experiment, sugar water and regular water were placed in two separate drinking bottles and labeled clearly. Drinking bottles of sugar and ordinary water were placed into the animal cage simultaneously so that the animal could freely choose to drink; consumption of both liquids over a specific period (e.g., 12 h or 24 h). Liquid consumption was measured by weighing or using a drinking bottle marked on a scale. Calculation of the Sugar Water Preference Index (SPI): SPI = (sugar water intake/total intake) × 100%, where total intake was the sum of sugar water intake and regular water intake.

### 3.16. Morris Water Maze (MWM)

Spatial learning and memory were assessed using the MWM test, which was conducted in a circular tank (120 cm in diameter) where diatomite was sprinkled to make the water turbid. A circular platform is placed in each quadrant. Each mouse was randomly placed in one of the quadrants and allowed to locate the platform for 3 min. If the mice failed to climb the platform within 3 min, it was guided to the platform and allowed to remain on the platform for 30 s. After 5 days of continuous training, the platform was removed during the formal experiment, and the mice were allowed to swim freely in the tank. The time, speed, and total path spent by each mouse in the target quadrant were recorded. All tracks were tracked and analyzed using a video tracking system (Smart 3.0, Panlab, Cornellà de Llobregat, Spain).

### 3.17. Open Field Test (OFT)

The open field test (OFT) is a commonly used animal behavior experiment. OFT can detect the spontaneous activity behavior and exploration behavior of rats or mice, and it is a method to evaluate the autonomous behavior, exploration behavior, and tension degree of experimental animals in a new environment. Mice were placed in an open, black, unobstructed cube device (80 cm in length, width, and height) divided into nine equal parts. Silence was maintained throughout the experiment, and mice were allowed to explore freely for 3 min. Their activities were observed. The camera above the device recorded and analyzed the mice’s immobility time.

### 3.18. 8-Arm Maze

The 8-Arm Maze consists of eight identical arms radiating from a central platform. After 1 week of adaptive training in an 8-arm maze, the mice fasted for 24 h. On the second day, food particles were scattered on each arm and the center of the maze. Simultaneously, multiple mice were placed in the center of the maze (the door leading to each arm was open) and allowed to eat freely and explore for 10 min. The training was repeated on the third day. From day 4 onward, mice were trained individually: a grain of rat food was placed on the outer end of each arm to allow the animal to eat freely. The mice were removed either after eating all of the food or 10 min later. The training was repeated twice a day on day 5. After the sixth day, four arms were randomly selected, one pellet was placed in each arm, the mice were placed in the center of the maze, and the mice were allowed to move freely in the maze and ingest pellets until the mice had eaten all the food in all four arms. The experiment was terminated when the particles were not consumed after 10 min. The software was used to record the activity of the mice in the arm and central areas where the rat food was placed.

### 3.19. Nissl Staining

After the behavioral experiments, blood was collected from the eyeballs of the mice. The mice were then sacrificed, fresh brain tissue was removed, fixed with paraformaldehyde fixing solution, and conventional dewatering and embedding were performed. Brain tissue sections were cut into 5 μm thick sections and dewaxed to water. The sections were placed in a toluidine blue staining solution and stained in a 30 °C incubator for 1 h. After washing with deionized water, the samples were quickly dehydrated using absolute ethanol, transparent xylene, and neutral gum sealing. The neuronal structure of the hippocampus was observed under a light microscope.

### 3.20. Transmission Electron Microscopy (TEM)

The hippocampus was dissected from fresh mouse brain tissue, cut into small pieces of 1 mm^3^, fixed in an electron microscope fixative solution at room temperature, and protected from light. The hippocampal tissue was then fixed in 1% osmic acid at room temperature for 2 h, dehydrated with an alcohol gradient, washed twice with 100% acetone for 30 min each time, permeabilized, polymerized, ultra-thin sectioned at 60–80 nm in an ultrathin microtome, stained with 2.6% lead citrate solution for 8 min, protected from carbon dioxide, and placed under a TEM to observe the mitochondrial structure and collect images for analysis.

### 3.21. Immunohistochemical Staining

The mouse brain tissue was embedded routinely and sliced into 5 μm thick sections. Hydration, dewaxing, and antigen retrieval were performed. Endogenous peroxidases were removed using 3% H_2_O_2_ and blocked. The blocking solution was decanted, and the primary antibody diluent was incubated overnight. After washing with PBS 3 times, the secondary antibody was incubated at room temperature for 30 min. DBA staining was followed by hematoxylin counterstaining. Finally, the immunohistochemical staining results of SIRT1 (1:150) and PGC-1α (1:200) positive cells in the hippocampus were calculated by ImageJ 2022 software, and the mean values were analyzed statistically.

### 3.22. Immunofluorescence

Mouse brain tissue sections were dewaxed, hydrated, and placed in antigen repair buffer for antigen repair; closed with 3% BSA at room temperature for 30 min; then incubated with primary antibody at 4 °C overnight. The sections were washed with PBS, incubated with the secondary antibody at room temperature in the dark for 50 min, and washed again with PBS. The brain tissue sections were incubated with DAPI staining solution in the dark for 10 min, the nuclei were re-stained, and the sections were sealed with anti-fluorescence quenching tablets. Finally, sections were observed under a fluorescence microscope, and images were collected. Nuclei stained with DAPI were blue under ultraviolet excitation light, DRP1-positive expression was indicated by green fluorescence, and TOMM20-positive expression was indicated by red fluorescence.

### 3.23. Enzyme-Linked Immunosorbent Assay (ELISA)

The mouse blood was centrifuged at 8000 rpm for 15 min at 4 °C, and the supernatant was collected. Serum 5-HT and MDA levels were measured using by ELISA immunosorbent assay. ELISA was performed according to the guiding principles of the ELISA kit and the method provided by the manufacturer. The absorbance of each well was measured using a microplate reader (BioTek Epoch 2, Bio Tek Instruments, Winooski, VT, USA) at the wavelength recommended by the manufacturer.

### 3.24. Western Blot Analysis

Total protein was extracted from hippocampus tissues and cells using standard protocols; protein quantity was detected by BCA protein quantification; samples were separated by SDS-PAGE (80 V, 0.5 h, then 120 V, 1 h), transferred to PVDF membrane (100 V, 1 h), blocked with skim milk powder for 2 h, incubated overnight with primary antibody diluent at 4 °C, then washed with TBST, and incubated with HRP-labeled goat anti-rabbit IgG (H + l) for 2 h at room temperature. After washing with TBST, the membrane was developed using an enhanced chemiluminescence detection kit, and the membrane was visualized using a Jena Multifunction Imager (analytik jena, Jena, Germany). The images were quantitatively analyzed using ImageJ 2022 software.

### 3.25. Statistical Analysis

All data and statistical analyses complied with the recommendations for the experimental design and pharmacological analysis. Statistical comparisons were performed using a one-way analysis of variance (ANOVA). Statistical significance was set at *p* < 0.05.

## 4. Discussion

In this study, we demonstrated that 20 (S)-Protopanaxadiol improved depression-like behavior and hippocampal neuronal damage in chronically stressed mice. Furthermore, we found that 20 (S)-Protopanaxadiol can regulate the expression of SIRT1 and PGC-1α, an important downstream protein of SIRT1, which is closely related to mitochondrial biogenesis and function. Therefore, we examined the morphology and function of hippocampal mitochondria in chronically stressed mice by transmission electron microscopy and associated kits and found that 20 (S)-Protopanaxadiol significantly restored hippocampal mitochondrial damage, ATP production, and excessive ROS accumulation induced by CUMS stimulation. Furthermore, because mitochondrial mitosis and fusion directly affect mitochondrial function, we examined the expression level of DRP1, a mitochondrial mitosis regulator, and its aggregation on the outer mitochondrial membrane. The results showed that 20 (S)-Protopanaxadiol significantly reduced the CUMS stimulation-induced increase in DRP1 levels and the surge in DRP1 aggregation into the mitochondrial outer membrane. Similarly, in vitro, 20 (S)-Protopanaxadiol protects hippocampal neurons (HT22) from cortisol-induced cell viability decline and apoptosis, modulates SIRT1, PGC-1α, and DRP1 expression, reduces mitochondrial division, inhibits ROS production, and restores mitochondrial function (mitochondrial ATP production and mitochondrial membrane potential increase). Further experiments showed that EX-527, a SIRT1 inhibitor, significantly inhibited the effects of 20 (S)-Protopanaxadiol on mitochondrial function in CUMS mice and HT22 cells.

The CUMS model is a widely used animal model of depression. They exhibit many core depressive behaviors, including helplessness, anhedonia, and reduced motor activity [36]. Consistent with other findings, CUMS decreased sucrose preference, indicating that mice developed anhedonia, a core symptom of depression [11], and 20 (S)-Protopanaxadiol increased sucrose preference. Fluoxetine, a commonly used clinical antidepressant, was used as a positive control. Fluoxetine protects astrocytes by promoting autophagosome formation and clearing damaged mitochondria in CMS mice [30]. We examined the behavioral ability of mice using MWM, OFT, and 8-arm maze. We found that 20 (S)-Protopanaxadiol improved spatial memory and learning ability [37], activity ability, and exploration ability in mice.

The underlying mechanisms that lead to neuronal degeneration and death, such as oxidative stress, mitochondrial aberrations, and inflammation, are considered important in the pathogenesis of a range of neurological diseases [38,39,40,41]. Repeated injections of cortisol in animals have been reported to cause hypothalamic axis abnormalities, neuronal damage, and the induction of depression-like behavior [42]. Therefore, we attempted to establish an in vitro model of depression by stimulating corticosterone in vitro. 20 (S)-Protopanaxadiol is an active metabolite of ginsenosides in the gut and has been reported to have various biological properties, including anti-inflammatory, antioxidant, and antitumor properties [43]. In previous studies, 20 (S)-Protopanaxadiol exerts antidepressant effects possibly by regulating the normalization of neurotransmitter and corticosterone levels, relief of oxidative stress, and PI3K/Akt/mTOR-mediated BDNF/TrkB pathway in chronic social failure stress (CSDS) mice [44]. In this study, network pharmacology and molecular docking results showed that 20 (S)-Protopanaxadiol had strong binding activity with SIRT1. SIRT1 is thought to increase mitochondrial health by activating PGC-1α, which is important for regulating mitochondrial biogenesis, particularly in the heart and brain [45]. We therefore examined the expression of SIRT1 and PGC-1α in the hippocampus of CUMS mice and CORT-induced HT22 cells.

Based on the association between spatial memory and the hippocampus, we studied hippocampal neurons and mitochondria (32). We found that 20 (S)-Protopanaxadiol improved hippocampal neuronal damage, neuronal apoptosis, and mitochondrial dysfunction. Mitochondria adapt to various stress conditions through continuous fusion and fission to meet cellular energy metabolism and other biological requirements. This biological process is known as mitochondrial dynamics [21]. Mitochondrial dynamics have been recognized as an important process leading to mitochondrial damage in various pathological states such as insulin resistance, obesity, neurodegenerative diseases, and cancer [35]. When mitochondrial fission increases, the mitochondria exhibit a granular structure and function is impaired [46]. When FIS1 reaches the mitochondrial outer membrane (OMM), it recruits DRP1 from the cytoplasm to aggregate in the OMM together with mitochondrial fission factors, forming oligomers that wrap around the OMM and apply mechanical force to destroy the OMM and initiate mitochondrial division [35,47]. Therefore, we detected DRP1 expression by Western blotting and observed DPR1 aggregation in the outer mitochondrial membrane by double immunofluorescence staining. 20 (S)-Protopanaxadiol reduced DRP1 expression, inhibited DRP1 aggregation in the outer mitochondrial membrane, and reduced mitochondrial division.

To investigate the role of SIRT1 in reducing mitochondrial fragmentation and improving mitochondrial function, we inhibited SIRT1 expression using the selective SIRT1 inhibitor, EX-527, in CUMS-treated mice and CORT-induced HT22 cells. Our results suggest that DRP1 expression and DRP1-mediated mitochondrial destruction can be attenuated by the application of 20 (S)-Protopanaxadiol, and upon application of EX-527, this inhibitor suppresses the expression of SIRT1, which in turn reduces the expression of PGC-1α. The expression level of DRP1 increased again and the protective effects of 20 (S)-Protopanaxadiol on mitochondrial morphology and function were greatly reduced. PGC-1α is a PPARγ receptor coactivator that regulates the biogenesis of new mitochondria and plays a decisive role in maintaining carbohydrate, lipid, and energy homeostasis in the body [48]. In addition, EX-527 inhibited SIRT1 expression and partially reversed the protective effect of 20 (S)-Protopanaxadiol against CORT-induced apoptosis and necrosis. In conclusion, our results provide clues for further elucidation of the regulatory mechanism of 20 (S)-Protopanaxadiol in mitochondrial division.

As described above, our in vitro and in vivo studies confirmed the important role of mitochondrial division in CUMS- and CORT-induced mitochondrial dysfunction and neural damage. 20 (S)-Protopanaxadiol ameliorates mitochondrial dysfunction and reduces excessive apoptosis through the SIRT1/PGC-1α signaling pathway. Furthermore, our study suggests that DRP1 may be a potential therapeutic target for depression and that 20 (S)-Protopanaxadiol may improve the symptoms of depression by modulating DRP1-mediated mitochondrial division. Furthermore, since abnormalities in mitochondrial dynamics provide clues to the potential novel mechanisms of neurological diseases [35], the property of 20 (S)-Protopanaxadiol to maintain mitochondrial kinetic equilibrium brings another way to its broad neuroprotective effects.

## 5. Conclusions

Our study suggests that 20 (S)-Protopanaxadiol may act as a SIRT1 activator to ameliorate CUMS- or CORT-induced depressive-like behaviors, neuronal damage, and apoptosis by activating the SIRT1/PGC-1α pathway, inhibit excessive accumulation of ROS, and promote mitochondrial ATP production. More importantly, we found that 20 (S)-Protopanaxadiol can improve mitochondrial dysfunction and DRP1-mediated mitochondrial division by promoting the expression of SIRT1. In conclusion, this study extends the pharmacological activity of 20 (S)-Protopanaxadiol in relieving depression and alleviating mitochondrial damage and provides a therapeutic strategy for 20 (S)-Protopanaxadiol in treating depression.

## Data Availability

The data that support the findings of this study are available from the corresponding author upon reasonable request.
